# Red Ginseng Oil Attenuates Oxidative Stress and Offers Protection against Ultraviolet-Induced Photo Toxicity

**DOI:** 10.1155/2021/5538470

**Published:** 2021-07-01

**Authors:** H. M. Arif Ullah, Yuan Yee Lee, Minki Kim, Tae-Wan Kim, Evelyn Saba, Yi-Seong Kwak, Mansur Abdullah Sandhu, Man Hee Rhee

**Affiliations:** ^1^Laboratory of Physiology and Cell Signaling, College of Veterinary Medicine, Kyungpook National University, Daegu 41566, Republic of Korea; ^2^Department of Veterinary Biomedical Sciences, Faculty of Veterinary and Animal Sciences, Pir Mehr Ali Shah-Arid Agriculture University, Rawalpindi, Pakistan; ^3^R&D Headquarters, Korea Ginseng Cooperation, Daejeon 34520, Republic of Korea

## Abstract

Ginseng (*Panax ginseng* Meyer) is a well-known herbal medicine that has been used for a long time in Korea to treat various diseases. This study investigated the *in vitro* and *in vivo* protective effects of red ginseng extract (RGE) and red ginseng oil (RGO). Liver injury was produced in BALB/c mice by 400 mg/kg of acetaminophen intraperitoneal injection. The antioxidant effects of RGE and RGO on the free radicals 2,2-diphenyl-1-picryl-hydrazyl-hydrate (DPPH) and 2,2′-azino-bis-3-ethylbenzothiazoline-6-sulfonic acid (ABTS) were measured. In addition, the hepatoprotective activities of RGE and RGO on liver markers, including alanine aminotransferase (ALT), aspartate aminotransferase (AST), and oxidative stress markers, including superoxide dismutase (SOD), catalase (CAT) enzyme activity, and 8-hydroxy-2-deoxyguanosine (8-OHdG) in serum and histopathological analysis, were evaluated. The protective effect of RGO on UV-induced phototoxicity was also evaluated in Balb/c 3T3 mouse fibroblast cell line. RGE and RGO effectively inhibited the radicals DPPH and ABTS compared with ascorbic acid and trolox, respectively. Moreover, RGE and RGO significantly decreased the liver enzyme (ALT and AST) levels, increased the antioxidant enzyme (SOD and CAT) levels, and decreased the DNA oxidation product (8-OHdG) content in mice serum. RGO also exhibited protective effect against UV irradiation compared with chlorpromazine hydrochloride, a known phototoxic drug, in Balb/c 3T3 cell line. RGE and RGO possess antioxidant and hepatoprotective properties in mice, and RGO exerts nonphototoxic activity in Balb/c 3T3 cells.

## 1. Introduction

Ginseng has been used as an herbal medicine and a functional food in Asia for more than 200 years due to its beneficial effects [1–3]. Approximately 12 species of ginseng have been recognized, among which *Panax ginseng* Meyer (Korean ginseng) is well-known and widely consumed. Red ginseng is the most widely used ginseng product in Asia, especially Korea, due to its various pharmacological activities, including antidiabetic, anticancer, antiplatelet, antioxidant, and antiobesity effects [4–7]. The major chemical compounds in red ginseng are the key sources for its medicinal activities [1]. Red ginseng is produced from fresh ginseng using a repeated process of steaming and followed by drying process [3, 8]. Ginseng is commonly consumed in the various forms including tablets, capsules, candies, jellies, and various types of fermented products [1].

Ginsenosides are the bioactive constituents of red ginseng and having biological properties. Various ginsenosides have been discovered in red ginseng extract (RGE) and used widely due to their specific pharmacological effects [9]. Studies reported that the major ginsenoside profiles of the RGE are Rg1, Re Rf, Rh1, Rg2, Rb1, Rc, Rb2, Rb3, Rd, and Rg3 [1, 10]. In addition, based on gas chromatography analysis, it has been shown that the major constituents of red ginseng oil (RGO) are unsaturated fatty acid (linoleic acid), saturated fatty acid (palmitic acid), phytosterins (alpha-tocopherol, Beta-sitosterol, gamma-sitosterol, Stigmasterol), and hydrocarbon (bicyclo) [11, 12]. It has been reported that red ginseng essential oil has antioxidant and hepatoprotective effects in hydrogen peroxide- (H_2_O_2_-) treated HepG2 cells and carbon tetrachloride- (CCl_4_-) treated mice [8]. Moreover, a recent study demonstrated the antimelanogenic activities of Korean red ginseng oil (RGO) in a UV-B-induced hairless mouse [13].

Oxidative stress is the result of the production of reactive oxygen species (ROS) and reactive nitrogen species generated by the normal metabolic process and by exogenous stimuli in the body. These free radicals can damage the structure and function of cells, and increased concentrations of free radicals in metabolism are responsible for the development of various diseases [14, 15]. Antioxidant enzymes and antioxidant molecules can prevent or reduce the generation of these free radicals through the antioxidative defense system, which neutralizes ROS or scavenges free radicals [14, 16]. Several previous studies have demonstrated that an overdose of acetaminophen (APAP) can increase ROS production, and as a result, the oxidative stress causes severe liver injury [17–19]. APAP induces oxidative stress that can attack cell organelles, damage the cell membrane, induce lipid peroxidation, and ultimately result in liver injury [20]. Furthermore, high-energy visible and ultraviolet (UVA specifically) light have high energy and cause strong phototoxic reaction [21, 22]. The phototoxic reaction is primarily induced by exposure of photoreactive chemicals to UV light, and these chemically activated compounds are responsible for the formation of ROS/free radicals [23]. Hence, UV can damage the skin cells, which may lead to aging and cause itchiness, wrinkles, pigmentation, erythema, and eschar formation [21, 22].

This study was conducted to determine antioxidant and hepatoprotective potential of red ginseng extract (RGE) and red ginseng oil (RGO) in mice and the nonphototoxic activity of RGO using the Balb/c 3T3 mouse cell line. Here, we demonstrated that RGE and RGO have potential scavenging effects on free radicals, thus suggesting an important role in oxidative stress.

## 2. Materials and Methods

### 2.1. Chemicals and Reagents

The following materials were used in this study: RGE and RGO (Korea Ginseng Corporation, Daejeon, Republic of Korea), chlorpromazine hydrochloride (CPZ; Sigma, #MKBT6268V), Balb/c 3T3 cells, clone A31 (ATCC), DMEM (Gibco, #2041847), newborn calf serum (Gibco, #1749272), penicillin/streptomycin (Gibco, #2019314), neutral red (NR) solution (Sigma, #RNBG6531), and UV lamp (BIO-SUN, Serial number, 15-101159). All other chemicals and reagents were of the highest grade.

### 2.2. Sample Preparation of RGE and RGO

Red ginseng powder was obtained from Korea Ginseng Corporation (Daejeon, Republic of Korea). RGE was prepared using water as described in a previously reported procedure with slight modification [24]. Briefly, in hot water, red ginseng was extracted and heated at 90°C for 1 h. After cooling, the supernatant was collected and centrifuged at 3000 rpm for 5 min and evaporated. After evaporation, the samples were stored at 4°C until use. RGO was prepared as described previously with modification [8]. Briefly, red ginseng powder was extracted with the CO_2_ extraction system at a pressure of 450 bar and a temperature of 65°C. The oil extract of red ginseng was preserved in a vial and stored at 4°C until use.

### 2.3. Cell Culture

Balb/c 3T3 mouse cells were cultured in DMEM supplemented with 10% newborn calf serum and 1% 100 IU/mL penicillin and 100 *μ*g/mL streptomycin. Cells were seeded in a 96-well plate at different densities for the measurement of cell viability. RGO concentrations of 7.81, 15.63, 31.25, 62.5, 125, 500, and 1000 *μ*g/mL were used. For positive control, CPZ concentrations of 0.04, 0.08, 0.16, 0.31, 0.63, 1.25, 2.5, and 5 *μ*g/mL and 1.56, 3.13, 6.25, 12.5, 25, 50, 100, and 200 *μ*g/mL were used with UV and without UV, respectively.

### 2.4. Irradiation Condition

A UV irradiation device (BIO-SUN, Serial number, 15-101159) was used for UV light source. The amount of UV light was 5 J/cm^2^ with emitting wavelengths of 315–400 nm. For this amount of UV light (5 J/cm^2^), the exposure time was calculated by applying the light intensity measured 1 min after the starting of UV irradiation.

### 2.5. Animal Treatment

The animals were housed in pathogen-free facility at 21 ± 2°C with a humidity of 60 ± 10% under a 12-h light and dark cycle and feed and water were supplied ad libitum. Animal care and experimental procedure were conducted with the Institutional Animal Care and Use Committee (IACUC) guidelines, and the animal protocols were approved by the Animal Care Committee of the College of Veterinary Medicine, Kyungpook National University, Daegu, South Korea (approval number: 2019-0046). Male Balb/c mice aged 6 weeks were treated with 100 mg/kg and 300 mg/kg of RGE and RGO for 2 weeks orally every day. As a positive control, 75 mg/kg N-acetyl cysteine (NAC) was used. After 2 weeks, the mice were injected with 400 mg/kg of APAP through the intraperitoneal route, and after 2 h, the mice were anesthetized, and blood was collected by cardiac puncture. The blood was left to settle for 2 h in a blood collection tube (BD, Plymouth, UK). The tubes were then centrifuged at 3000 rpm for 15 min to separate the serum, which was collected and stored at −70°C until assay. Liver sample was collected for histopathological examination.

### 2.6. Measurement of Antioxidant Activity

The DPPH free-radical scavenging activity and 2, 2′-azino-bis-3-ethylbenzothiazoline-6-sulfonic acid (ABTS) radical-scavenging activity of RGE and RGO were evaluated using the method described by Kandi Sridhar and Albert Linton Charles with modification [14]. Briefly, RGE and RGO sample extract or positive control (ascorbic acid) with various concentrations were added to the same volume of DPPH (100 *μ*M). The mixtures were vortexed and incubated at room temperature for 20 min in the dark. Finally, the absorbance was determined using a spectrophotometer at 515 nm. For the ABTS assay, the ABTS method was produced with reacting the aqueous solution of ABTS with potassium persulfate in the same volume and allowed to react for 24 h at room temperature in the dark. The ABTS radical cation (1 mL) solution was mixed with 0.1 mL of extract or positive control (trolox) at different concentrations. The mixtures were incubated at room temperature for 10 min in the dark. Finally, the absorbance was measured at 734 nm with spectrophotometer.

### 2.7. Measurement of Serum Markers for Oxidative Stress Alanine Aminotransferase and Aspartate Aminotransferase Activity in Serum

Mice were sacrificed 2 h after the last administration from each group. Blood samples were collected and centrifuged. The levels of alanine aminotransferase (ALT), aspartate aminotransferase (AST), superoxide dismutase (SOD), catalase (CAT), and 8-hydroxy-2-deoxyguanosine (8-OHdG) were measured according to kit instructions.

### 2.8. Histological Analysis

For evaluating the histopathological changes, the liver tissues were fixed in 10% formalin and routinely processed in a graded ethanol series and toluene, as described previously [25]. The tissues were then embedded in paraffin and sectioned into 5-*μ*m-thick slices. The sections were stained with hematoxylin and eosin (H&E) as previously described, and the pathological changes were observed under a light microscope.

### 2.9. Determination of Phototoxicity

Phototoxicity was evaluated using RGO and CPZ on the mouse fibroblast cell line Balb/c 3T3 using a previously described method [21]. Cells were seeded in two 96-well plates with (+UV) and without (−UV) UV light for RGO and CPZ at a density of 1 × 10^4^ cells/well. DMSO in cell media was used as a negative control. After dispensing 100 *μ*L of cell suspension (1 × 10^4^ cells/well) in the culture medium, the plates were incubated for 24 h at 37°C and 5% CO_2_. After 24 h, the medium was removed, cells were washed with PBS, and 100 *μ*L of the test materials, positive control, and negative control were added to the wells (*n* = 3). The plates were incubated at 37°C and 5% CO_2_ for 1 h, after which the irradiated plate was exposed to UV light (5 J/cm^2^), and the nonirradiated plate was shielded with an aluminum foil. After UV exposure, the medium was decanted, and 100 *μ*L of the medium was added. The cells were incubated at 37°C and 5% CO_2_ for 24 h. The medium was removed and washed with PBS. Then, 100 *μ*L of NR solution (50 *μ*g/mL) was added to each well and incubated at 37°C and 5% CO_2_ for 3 h. After removing the NR medium, it was washed with PBS. Next, 150 *μ*L of NR extractant solution was added to each well, and the plates were shaken on a plate shaker for 30 min in a light-shielded state. Finally, the absorbance was measured at a wavelength of 540 nm using a multichannel microplate reader. The absorbance data were used in the phototox software (Phototox 2.0, ZEBET at the BFR, Berlin Germany) to obtain cell viability data at different concentrations.

### 2.10. Statistical Analysis

The experimental data were expressed as mean ± the standard deviation (SD). Statistical significance was determined using ANOVA. The statistical significance of data is denoted on the graphs by asterisks (^∗^), with the significance values as ^∗^*P* < 0.05, ^∗∗^*P* < 0.01, and ^∗∗∗^*P* < 0.001.

## 3. Results

### 3.1. Effects of RGE and RGO on DPPH and ABTS Free Radicals

**Figure 1:**
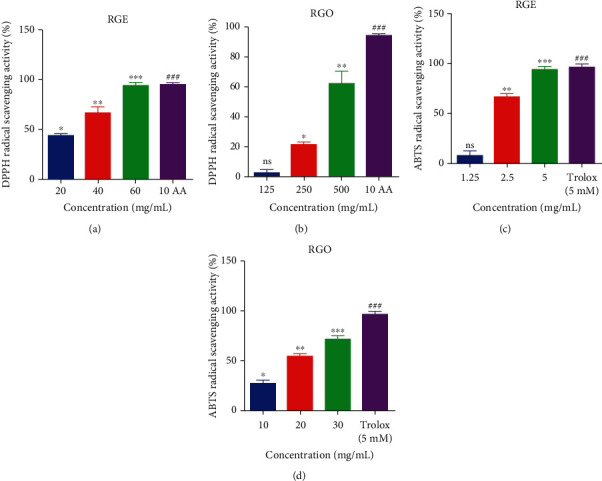
Determination of in vitro antioxidant activities of red ginseng extract (RGE) and red ginseng oil (RGO). (a, b) DPPH and (c, d) ABTS assays for RGE and RGO, respectively. In DPPH and ABTS methods, the standards were ascorbic acid (AA) in DPPH and trolox in ABTS, respectively. Radical-scavenging capacities were dose-dependent upon treatment with RGE and RGO. Data are presented as mean ± standard deviation (SD) (*n* = 3). ^###^*P* < 0.001 for standard, ^∗^*P* < 0.05, ^∗∗^*P* < 0.01, and ^∗∗∗^*P* < 0.001 compared with standard.

The antioxidant activities of RGE and RGO were determined using the DPPH and ABTS assay. The results showed that RGE effectively scavenged the radicals of DPPH at 60 mg/mL to the extent of the positive control, which was 10 mg/mL of ascorbic acid (Figure 1(a)). On the other hand, RGO scavenged the radicals of DPPH from concentrations of 125 to 500 mg/mL (Figure 1(b)). Regarding the ABTS assay results, it was found that RGE effectively reduced the radicals completely at 5 mg/mL concentration, similar to the level of trolox, a water-soluble conjugate of vitamin E that was used as a positive control in this assay (Figure 1(c)). The concentration of trolox used in this study was 10 *μ*M. In the case of RGO, a concentration of 30 mg/mL showed an efficacy of approximately 80% in inhibiting ABTS radicals (Figure 1(d)).

### 3.2. Effects of RGE and RGO on ALT and AST Activity in Serum

**Figure 2:**
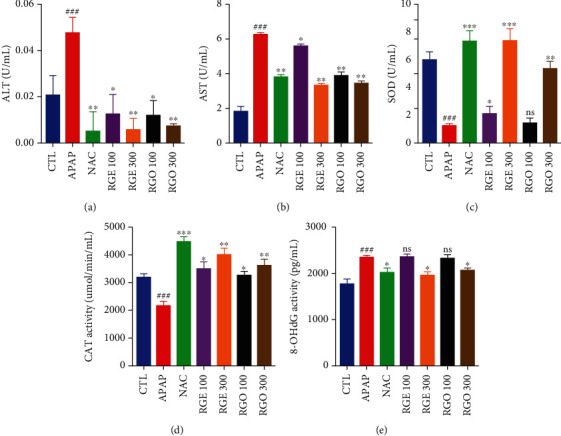
Effects of RGE and RGO in serum markers on APAP-induced liver injury in mice. (a) Effects of RGE and RGO on the ALT level in the serum. (b) Effects of RGE and RGO on the AST level in the serum. (c) Effects of RGE and RGO on the SOD activity in the serum. (d) Effects of RGE and RGO on the CAT activity in the serum. (e) Effects of RGE and RGO on the 8-OHdG content in the serum. Data are presented as mean ± standard deviation (SD) (*n* = 5). Significant difference at ^###^*P* < 0.001 compared with control (CTL) group and ^∗^*P* < 0.05, ^∗∗^*P* < 0.01, and ^∗∗∗^*P* < 0.001 compared with the APAP-treated group.

APAP treatment resulted in extremely elevated levels of ALT in the serum of mice compared to the control group. However, this increase was significantly reduced by NAC treatment. Treatment with RGE and RGO also reduced the levels of ALT in a dose-dependent manner (Figure 2(a)). NAC treatment reduced the levels of AST in serum induced by APAP. Treatment with 100 mg/kg RGE did not significantly reduce the levels of AST. However, treatment with 300 mg/kg RGE significantly reduced the levels of AST (Figure 2(b)). Comparatively, both concentrations of RGE and RGO led to a significant reduce in ALT and AST levels comparable to the levels observed with NAC treatment.

### 3.3. Effects of RGE and RGO on Oxidative Stress in Serum

SOD is an enzyme known to catalyze dismutation of the superoxide (O_2_^−^) radical into oxygen and hydrogen peroxide, which comprises an extremely important defense against oxidative stress in the body. Our results showed that APAP considerably reduced the levels of SOD in the serum. This reduction was recovered by NAC treatment. Treatment with 100 mg/kg of both RGO and RGE showed no significant increase in SOD levels. However, high doses of both RGO and RGE led to recovery of SOD levels (Figure 2(c)). CAT is an enzyme that is detected in all organisms when exposed to oxygen as it catalyzes the decomposition of hydrogen peroxide to water and oxygen. Therefore, it is an important enzyme against oxidative stress. Our study results showed that APAP reduced the activity of CAT compared to that in the control group. NAC treatment improved CAT levels in the serum to even higher levels than those in the control group. Both RGO and RGE increased the CAT activity in a dose-dependent manner (Figure 2(d)). 8-OHdG is the major product of DNA oxidation and is a marker of DNA damage. Our results demonstrated that in mice treated with APAP, 8-OHdG activity was increased and improved with NAC treatment. Treatment with RGE and RGO also improved the activity of 8-OHdG in a dose-dependent manner, where treatment with 300 mg/kg of both samples recovered the DNA oxidative damage to the levels of the control group (Figure 2(e)). These results may be due to the presence of the major ginsenosides in red ginseng determined by ultraperformance liquid chromatography (UPLC) (Table 1).

### 3.4. Effects of RGE and RGO on Pathological Changes in the Liver

**Figure 3:**
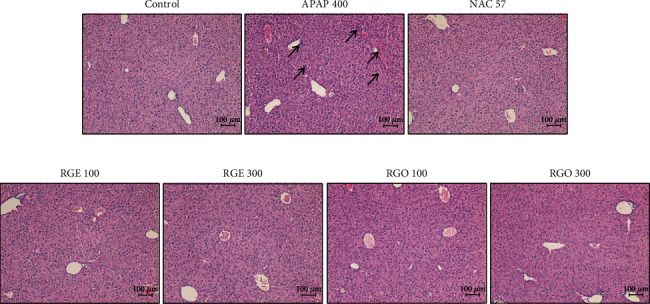
Histopathological changes by RGE and RGO on APAP-induced liver injury in mice. Mice were treated with APAP (400 mg/kg) to induce hepatotoxicity. In the APAP-treated group, there were degeneration of hepatocytes, congestive and dilated central veins, infiltration of inflammatory cells, vacuolation of hepatocytes, and necrosis of hepatocytes (arrows). N-acetyl cysteine (75 mg/kg) was used as a positive control. The concentrations of 100 and 300 mg/mL were used for RGE and RGO, respectively. Original magnification ×100.

Hepatocyte cells showed normal morphology, the hepatic lobules and sinusoids were intact, and the central veins were normal in the control group. However, in the APAP-treated group, the central veins were congestive and dilated, and degeneration of hepatocytes, infiltration of inflammatory cells, vacuolation of hepatocytes, and necrosis of hepatocytes were observed. In the NAC-treated group, there were no histopathological changes. In addition, treatment with 100 mg/kg of RGE and RGO significantly recovered the tissue damages compared to the APAP-treated group. Moreover, groups treated with 300 mg/kg of RGE and RGO showed normal arrangement of hepatocytes with no significant damage and infiltration of inflammatory cells and hepatocytes, similar to that in the control group (Figure 3). This result indicated that RGE and RGO have significant protective effects against APAP-induced hepatic injury.

### 3.5. Effects of RGO on Phototoxicity

**Figure 4:**
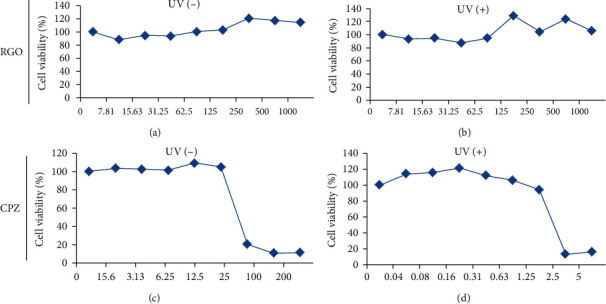
Effects of RGO and CPZ on cell viability with UV (-) and UV (+) light exposure in Balb/c 3T3 cells. (a, b) Cell viability was evaluated at different concentrations of RGO in the absence or presence of UV light. (c, d) Cell viability was evaluated at different concentrations of chlorpromazine (CPZ) in the absence or presence of UV light.

**Figure 5:**
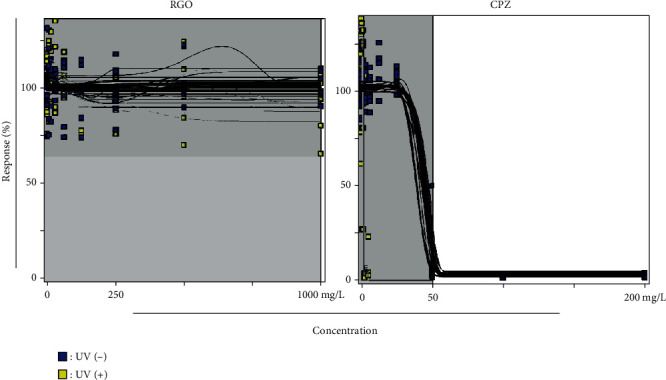
Effects of concentrations in RGO and CPZ with UV (-) and UV (+) light exposure in Balb/c 3T3 cells. (a) Effect of increasing concentrations with UV (-) and UV (+) of RGO on percentage response of Balb/c 3T3 cells. (b) Effect of increasing concentrations with UV (-) and UV (+) of CPZ on percentage response of Balb/c 3T3 cells. No exposure of UV light (blue) and exposure to UV light (yellow).

Phototoxicity was evaluated using RGO and CPZ in Balb/c 3T3 mouse cell line. Cell viability was measured at various concentrations of RGO and CPZ in the presence (exposure) or absence (no exposure) of UV light (Figures 4(a)–4(d)). RGO was found to be nonphototoxic and displayed insensitivity to phototoxic reaction (Figure 5(a)), whereas CPZ demonstrated toxicity potential in the presence of UV light (Figure 5(b)). Earlier, Peters and Holzhutter (2002) reported that sample is considered to be nonphototoxic if it has a photoirritation factor (PIF) < 2 or a mean photo effect (MPE) < 0.1; probably phototoxic if PIF is >2 and <5 or MPE is >0.1 and <0.15; and phototoxic if PIF is >5 or MPE is >0.15. In the case of CPZ, the PIF and MPE values were 25.721 and 0.236, respectively. Based on the assessment of parameters, RGO was found to be completely nonphototoxic, with the MPE being 0.006 (MPE < 0.1, nonphototoxic) [26]. In contrast, CPZ exhibited phototoxic activity, with the MPE being 0.236 (MPE > 0.15, phototoxic), in the presence of UV light (Table 2).

## 4. Discussion

The liver is the major organ that metabolizes the majority of drugs. APAP is one of the most widely used drugs to reduce fever and pain. However, an overdose of APAP induces oxidative stress and production of ROS, which causes metabolic dysfunction, damage to the antioxidant defense system, and other tissue and hepatic injuries [20, 27]. Similarly, when cells are continuously exposed to sunlight, free radicals such as superoxide anion and hydroxyl radical and nonradical intermediates, including hydrogen peroxide and singlet oxygen, are generated [21]. These ROS can be produced by several sources, such as water, molecular oxygen, and enzymes. However, cells react to these ROS using nonenzymatic antioxidants such as vitamin C, vitamin E, and enzymatic antioxidants such as CAT and SOD [28]. Imbalance between these systems can modulate protein function, causing destructive action on DNA, which has been implicated in mutagenesis, carcinogenesis, and aging [21, 29]. Therefore, the inhibition of oxidative stress is a promising therapeutic strategy for liver damage and phototoxicity.

Several studies have demonstrated that natural antioxidants have potential pharmacological activities to protect organs from toxic substances, including the adverse effects of APAP and sunlight. The major active components in the RGE are the ginsenosides which play key role for the various beneficial effects [1]. Previous study demonstrated that sitosterol was influenced the cellular protective systems via enhancement of nuclear factor erythroid-2 related factor-2 (Nfr2) pathway in HepG2 cells [30]. Moreover, beta-sitosterol and stigmasterol were protected oxidative stress induced by nitrophenol through activation of Nrf2-mediated antioxidant enzymes in rat, reported by Zhang et al. [31].

In this study, the antioxidant properties of RGE and RGO were investigated using the commonly used antioxidant methods, including DPPH and ABTS assays. The results clearly demonstrated that both RGE and RGO significantly inhibited the production of free radicals based on the concentration obtained by DPPH and ABTS methods (Figures 1(a)–1(d)). The scavenging capacity of DPPH increased in a dose-dependent manner (20, 40, and 60 mg/mL and 125, 150, and 500 mg/mL for RGE and RGO, respectively). Ascorbic acid was used as a standard solution (10 mg/mL) that exhibited the highest inhibition (95.25%) by the DPPH method, whereas the highest inhibition value was 94.13% for RGE at the concentration 60 mg/mL. Similarly, with RGO treatment, the maximum inhibition was 62.64% at the highest concentration of 500 mg/mL. The DPPH scavenging capacity results showed that RGE and RGO exhibited high antioxidant effects compared with ascorbic acid. Ryu et al. reported about the free radical-scavenging capacity of Korean red ginseng for erectile dysfunction in rats with noninsulin-dependent diabetes mellitus [5].

On the other hand, a similar trend was observed by the ABTS method, wherein the scavenging activity of ABTS was increased in a dose-dependent manner (1.25, 2.5, and 5 mg/mL and 10, 20, and 30 mg/mL for RGE and RGO, respectively). This result was compared with the standard solution trolox (5 mM), a vitamin E conjugate, which showed the highest inhibition (97.25%). RGE treatment resulted in the highest inhibition value (95.14%) at the highest concentration (5 mg/mL), whereas RGO treatment also exhibited the same trend of the highest inhibition (72.7%) at the highest concentration (30 mg/mL). This result indicated that both RGE and RGO possess potent antioxidant capacity as evaluated by the ABTS assay. Earlier report demonstrated that ethanolic extracts of red ginseng and puffed red ginseng significantly showed radical scavenging ability by DPPH and ABTS assays [3].

Liver enzymes, primarily ALT and AST, in the blood are the major indicators to evaluate liver function [32]. Increasing levels of serum liver enzymes indicate hepatic injury because these enzymes are normally located in the cytoplasm, but after hepatic dysfunction, the enzymes are released into the circulation. An overdose of APAP increases the levels of liver enzymes, indicating liver damage. Kim et al., reported that pretreatment with Korean red ginseng reduced the expression of ALT and AST levels on aflatoxin B1-induced liver toxicity in rat model [33]. Our results demonstrated that serum ALT and AST levels were significantly reduced by RGE and RGO treatment (Figures 2(a) and 2(b)), suggesting that both RGE and RGO have protective activity against the APAP-induced liver injury.

SOD and CAT are the most important antioxidant enzymes against ROS. Under the normal physiological condition, SOD and CAT counteract with free radicals, but these enzymes are reduced when there is overproduction of ROS [34–36]. In the past, it was reported that saponin fraction of red ginseng supplementation was markedly increased SOD and catalase levels in hepatic tissue in mice [37]. In this study, the levels of SOD and CAT were significantly reduced after APAP administration; meanwhile, RGE and RGO treatment significantly elevated their activities (Figures 2(c) and 2(d)). These results indicated that both RGE and RGO significantly elevated the levels of SOD and CAT in mice serum. Our results supported the previous report which stated that RGE significantly upregulated the SOD activity in healthy subjects (age 20-65 years) [38].

8-OHdG is the major product of DNA oxidation, and its elevation in the serum indicates excessive oxidative stress [34, 39]. An overdose of APAP increased the DNA oxidation and reduced the activity of antioxidant enzymes. Our results demonstrated that the levels of 8-OHdG were significantly reduced in the RGE- and RGO-treated groups (Figure 2(e)), suggesting that both RGE and RGO could protect DNA oxidation and improve the DNA damage induced by APAP. Red ginseng extract showed potential antioxidant activity in oxidative stress-mediated DNA damage, various studies previously reported [33, 40].

Liver histological pathology is another indicator to evaluate hepatic injury [32, 41]. Observation of the hepatic tissue histopathology confirmed the inhibitory effects of RGE and RGO in APAP-treated liver damage. According to the H&E staining result, the RGE- and RGO-treated groups showed reduced hepatocyte degeneration and decreased infiltration of inflammatory cells compared to the APAP-treated group (Figure 3). Moreover, significantly reduced hepatic necrosis and dilated sinusoids were observed in the RGE- and RGO-treated groups. This protective activity was dose-dependent, although there was no high difference between two doses (100 and 300 mg/mL) in these groups. Based on histopathological findings, there was no significant difference between NAC treatment at 75 mg/mL and the control group. Pretreated with Korean red ginseng were significantly suppressed hepatic lesions such as necrosis of hepatocytes, hemorrhage, and loss of hepatic cords according to previous report [33]. Our results demonstrated that both RGE and RGO have significant protective capacity against APAP-induced hepatic damage.

In this study, we demonstrated RGO provides protection against UV light in Balb/c 3T3 cells (Figure 5(a)), suggesting that RGO has nonphototoxic capacity. It is assumed that the nonphototoxic activity could be caused because of antioxidant properties. CPZ was used as a positive control due to the phototoxic activity (Figure 5(b)) and caused damage to the DNA by direct and indirect mechanisms [21, 42]. However, further studies are required to confirm the degree of nonphototoxic activity of RGO.

## 5. Conclusions

**Figure 6:**
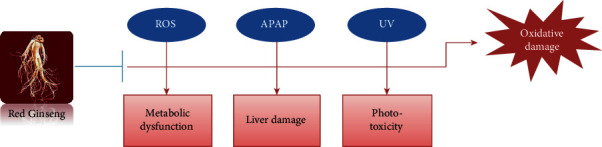
Schematic model showing the role of red ginseng in oxidative damage. Free radicals, overdose of acetaminophen (APAP), and UV radiation lead to metabolic dysfunction, liver toxicity, and phototoxicity, causing damage to the antioxidant defense system. Red ginseng extract (RGE) and red ginseng oil (RGO) protect against reactive oxygen species (ROS) and APAP- and UV-induced oxidative damage.

In summary, RGE and RGO exhibited antioxidant and radical-scavenging properties, suggesting that both RGE and RGO possessing antioxidant activities can prevent the cell or tissue from oxidative damage induced by toxic chemicals or radiation (Figure 6). Our study indicated that Korean red ginseng may be potential substance in skin care and sunscreen formulations. To the best of our knowledge, this is the first report to demonstrate the effects of RGE and RGO on oxidative stress and attenuated activity of RGO on ultraviolet-induced photo toxicity. Further study in large scale is mandatory to determine the exact mechanism underlying the protective effects of red ginseng.

## Figures and Tables

**Table 1:** Determination of ginsenosides in red ginseng [1]. Data are presented as mean ± standard deviation (SD) (*n* = 3).

Classification	Ginsenoside	Composition (mg/g)
Panaxatriol	Rg1	3.00 ± 0.033
	Rg2	3.26 ± 0.085
	Rg3	4.04 ± 0.049

Panaxadiol	Rb1	10.51 ± 0.048
	Rb2	4.51 ± 0.053
	Rb3	0.32 ± 0.067
	Rc	5.37 ± 0.074
	Rd	3.47 ± 0.111
	Rk1	4.04 ± 0.051

**Table 2:** Using the software (Phototox 2.0, at the BFR, Berlin Germany), photo irritation factor (PIF), and mean photo effect (MPE) values were generated for RGO and CPZ in Balb/c 3T3 cells.

Group	PIF	MPE	Phototoxicity	Phototoxic parameter
RGO	0	0.006	Nonphototoxic	PIF < 2 or MPE < 0.1 = nonphototoxic
CPZ	25.721	0.236	Phototoxic	PIF > 2 and <5 or MPE > 0.1 and <0.15 = probably phototoxic
				PIF > 5 or MPE > 0.15 = phototoxic

## Data Availability

Data analyzed or generated during this study are included in this manuscript.
